# Developmental auditory exposure shapes responses of catecholaminergic neurons to socially-modulated song

**DOI:** 10.1038/s41598-018-30039-y

**Published:** 2018-08-06

**Authors:** Helena J. Barr, Sarah C. Woolley

**Affiliations:** 1 Integrated Program in Neuroscience, McGill University, Montreal, QC Canada; 2 Center for Research on Brain, Language, and Music, McGill University, Montreal, QC Canada; 3 Department of Biology, McGill University, Montreal, QC Canada

## Abstract

Developmental sensory experience is critical to the tuning of sensory systems and can shape perceptual abilities and their neural substrates. Neuromodulators, including catecholamines, contribute to sensory plasticity in both older and younger individuals and provide a mechanism for translating sensory experience into changes in brain and behavior. Less well known, however, is whether developmental sensory experience has lasting effects on the neuromodulatory neurons themselves. Here, we used female zebra finches to investigate the degree to which developmental auditory experience can have lasting effects on the density and sensory responsiveness of catecholamine-synthesizing neuron populations. We found that hearing courtship, but not non-courtship, song increased expression of the activity-dependent immediate early gene cFOS in dopamine neurons of the caudal ventral tegmental area (VTA) and this increase was dependent on whether females heard adult song during development. Developmental song exposure also affected the density of dopamine producing neurons in the rostral VTA. In contrast, song-evoked responses in noradrenergic neurons of the Locus Coeruleus were not affected by either developmental song exposure or the social context of the stimulus. These data highlight the lasting effects that developmental auditory experience can have in shaping both the density and sensory responsiveness of dopamine neuron populations.

## Introduction

Developmental auditory experience can shape auditory abilities and their neural substrates^1–6^. For example, musical training during childhood results in dramatic structural changes in the brain, including in primary auditory areas, as well as improved melodic and rhythmic discriminations^1^. Similarly, exposing rats to specific stimuli, such as single frequency tones, during a ‘critical period’ in development results in increased cortical representations of the stimulus frequency^6^ as well as altered pitch discrimination^7^. Songbirds, such as the zebra finch, provide an ideal model to study how developmental auditory experience shapes neural phenotypes important for perception^5,8–11^. Male zebra finches produce complex, learned vocalizations (‘songs’) that are used by females to identify individuals and select mates. Both male and female song perception is shaped by developmental auditory experience (reviewed in^5,8^). For example, relative to normally-reared females, females reared without exposure to adult male song (‘song-naïve’) do not show consistent preferences for high-performance female-directed, courtship song renditions (FD song) over non-courtship, undirected renditions (UD9) despite demonstrating species-typical preferences for conspecific over heterospecific songs^10^. Moreover, differences in perception between normally-reared and song-naïve birds are evident in the song-evoked activity of forebrain auditory regions^9,11,12^.

Neuromodulators, such as dopamine (DA) and norepinephrine (NE), have been implicated in sensory cortical plasticity in adults^13,14^ as well as in developing animals^15^. In particular, both dopaminergic and noradrenergic neurons respond to salient or preferred stimuli, indicating to the brain which stimuli are important^16–22^. For example, in juvenile male zebra finches, NE and DA neurons are more active following social tutoring, which elicits better learning outcomes, than passive tutoring^23^. Forebrain auditory areas, such as the NCM, receive catecholaminergic projections, as indicated by the expression in fibers and terminals of tyrosine hydroxylase (TH), the rate-limiting enzyme in the synthesis of dopamine and norepinephrine^24,25^. Song responses in the NCM are modulated by NE in zebra finches, and manipulation of NE levels can reduce copulation solicitation displays to sexually stimulating songs, slow the learning of song discrimination, and reduce responses of forebrain auditory regions to conspecific song^26–30^. Thus, catecholamines may provide a mechanism for translating auditory experience into changes in brain and behavior. However, little is known about the degree to which sensory experience, especially during development, may affect the abundance or activity of neuromodulatory neurons themselves.

Here, we investigated the degree to which developmental song exposure shapes the density and activity of catecholamine populations in the midbrain and hindbrain. In particular, we quantified the number of catecholamine producing neurons as well as their expression of the immediate early gene protein (IEG) cFOS in the midbrain and hindbrain following playback of FD and UD song in normally-reared and song-naïve females. We found that in the ventral tegmental area (VTA), but not the locus coeruleus (LC), substantia nigra pars compacta (SNc), or periaqueductal gray (PAG), the number of catecholamine producing neurons as well as the modulation of cFOS expression within those neurons in response to social context were significantly influenced by developmental song exposure. These data highlight that developmental auditory experience can shape the responses of neuromodulatory systems to socially significant changes in communication signals.

## Results

**Figure 1:**
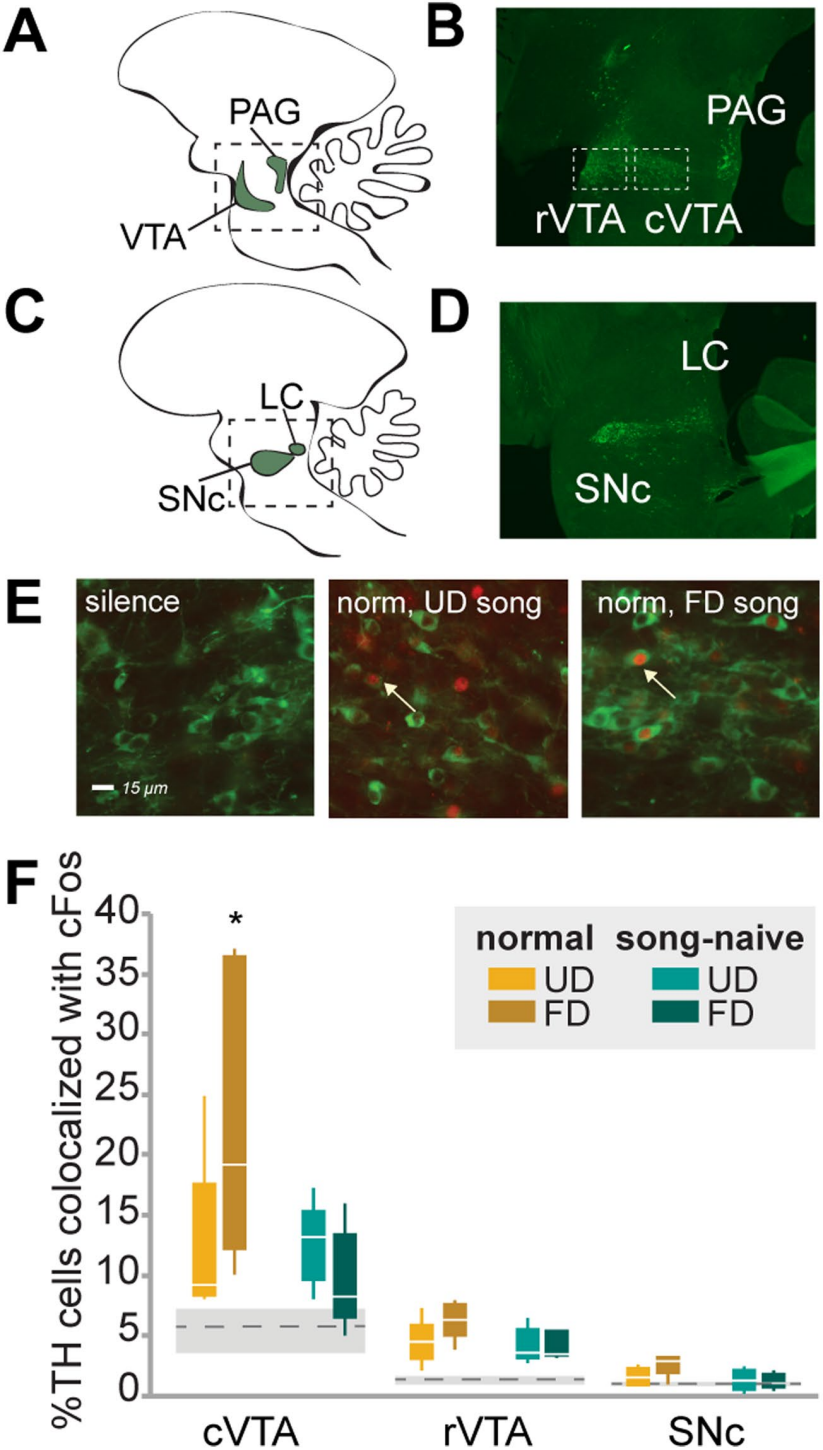
Developmental song exposure and social context affect cFOS expression in TH neurons of the caudal VTA. Sagittal drawings (**A**,**C**) and photomicrographs of tyrosine hydroxylase (TH; green label) expression (**B**,**D**) in the ventral tegmental area (VTA) and periaqueductal gray (PAG; panels A,B), and the substantia nigra pars compacta (SNc) and locus coeruleus (LC; panels C,D). (**E**) Examples of co-localized expression of TH (green) and cFOS (red) in the caudal VTA of a normally-reared bird that heard silence (left panel), UD song (middle panel; UD song) and FD song (FD; right panel). White arrows indicate examples of colocalized expression. (**F**) Percent of TH neurons co-localized with cFOS in the caudal VTA (cVTA), rostral VTA (rVTA) and SNc. Box-and-whisker plots for normally-reared (yellow colors) and song-naïve (green colors) hearing UD (UD; lighter colors) or female-directed (FD; darker colors) songs. Each box spans the interquartile range, horizontal white lines indicate the median and whiskers show the minima and maxima. Levels of cFOS expression in TH neurons for silence controls are plotted as the mean (gray dashed line) +/− standard error (gray boxes). ^*^Indicates a significant difference at p < 0.05 for all comparisons within a brain area.

Developmental auditory experience can shape the abundance and plasticity of cortical neurons, however it is unclear whether such experiences also have lasting effects on the abundance or activity of neuromodulatory populations that project to the cortex. Here, we investigated whether hearing adult song during development affected the song-evoked activity or number of catecholamine neurons in the rostral and caudal ventral tegmental area (rostral VTA; caudal VTA; Fig. 1A,B), the substantia nigra pars compacta (SNc; Fig. 1C,D), the locus coeruleus (LC; Fig. 1C,D) and the periaqueductal gray (PAG; Fig. 1A,B).

### Hearing female-directed song drives cFos expression in caudal VTA TH neurons, but only in normally-reared females

Dopamine neurons in the VTA are significant in encoding incentive salience, identifying important sensory stimuli, and shaping motor and cognitive responses^19,31^. We found that cFOS expression in the VTA was song-responsive. Specifically, hearing song significantly increased cFOS expression in tyrosine hydroxylase-immunoreactive (TH-ir) neurons in both the caudal VTA (F_(1,25)_ = 6.44, p = 0.0188; Fig. 1A,B,F; Table 1) and rostral VTA (F_(1,25)_ = 27.93, p < 0.0001; Fig. 1A,B,F; Table 1).

**Table 1:** Song playback elicits cFOS expression in TH neurons of the midbrain and hindbrain.

	cVTA	rVTA	SNc	LC	PAG
silence	5.27 +/− 0.60	1.27 +/− 0.17	1.03 +/− 0.10	10.26 +/− 1.62	20.92 +/− 3.60
sound	14.14 +/− 1.53^*^	4.80 +/− 0.31^*^	1.71 +/− 0.17^^^	36.21 +/− 2.16^*^	29.76 +/− 2.57

The percent of TH neurons colocalized with cFOS were significantly higher following song playback compared to silence in the cVTA, rVTA and LC (^*^indicates p < 0.05). In the SNc, there was a trend toward higher cFOS expression in TH neurons of the SNc (^^^p < 0.10). In the PAG, there was no significant difference in the colocalization of cFOS between birds that remained in silence and those that heard song playback.

While both rostral and caudal VTA exhibited song-evoked responses, only in the caudal VTA did rearing condition and stimulus context affect song responses. There was a significant interaction between stimulus, rearing, and region (F_(1,48.8)_ = 7.9, p = 0.0073). In normally-reared, but not song-naïve, females, cFOS expression in TH neurons in the caudal VTA was differentially affected by the social context of the stimulus. Specifically, the percent of TH neurons expressing cFOS in normally-reared females was significantly higher following playback of FD than UD song (p = 0.0152, Fig. 1E,F). Moreover, cFos expression in TH+ cells in the caudal VTA was significantly higher in normally-reared females that heard FD song than in song-naive females that heard either FD song (p = 0.0016) or UD song (p = 0.0233; Fig. 1F). However, there were no differences in the percent of TH+ neurons expressing cFOS in the in caudal VTA among either normally-reared or song-naïve females that heard UD song (p > 0.10). In addition, there were also no significant effects of either rearing or stimulus on cFos expression in TH+ neurons within the rostral VTA (p > 0.05 for all comparisons; Fig. 1F). We also imaged the caudal VTA using a confocal microscope and found no significant differences between counts of single images and confocal stacked images (t_(1,18)_ = −1.3, p = 0.2282; Supplementary Fig. S1). As was the case for the single image counts, there was a significant interaction between rearing and stimulus on the percent colocalization in a confocal image stack (F_(1,11.7)_ = 7.2, p = 0.0201; Supplementary Fig. S1) where cFOS expression was significantly higher in TH neurons of normally-reared females listening to FD song compared to normally-reared females listening to UD song (p = 0.0320), or song-naïve females listening to either FD song (p = 0,0043) or UD song (p = 0.0089). The increased cFOS expression in TH neurons in the caudal VTA of normally-reared females in response to FD song mirrors behavioral preferences for song, where normally-reared but not song-naïve females consistently prefer FD over UD song.

We also looked at the degree of cFOS expression in two additional midbrain dopaminergic regions: the substantia nigra pars compacta (SNc) and the periaqueductal gray (PAG). Dopaminergic neurons of the SNc are significant in shaping motor and cognitive responses through projections to the basal ganglia^32–34^. In the SNc, we found there was a trend for greater cFOS expression in birds in song-exposure conditions compared to birds in the silence condition (F_(1,25)_ = 3.19, p = 0.0862; Fig. 1F; Table 1). However, there were no significant effects of rearing condition, stimulus, or the interaction on the degree of cFOS-TH colocalization (p > 0.05 for all comparisons).

**Figure 2:**
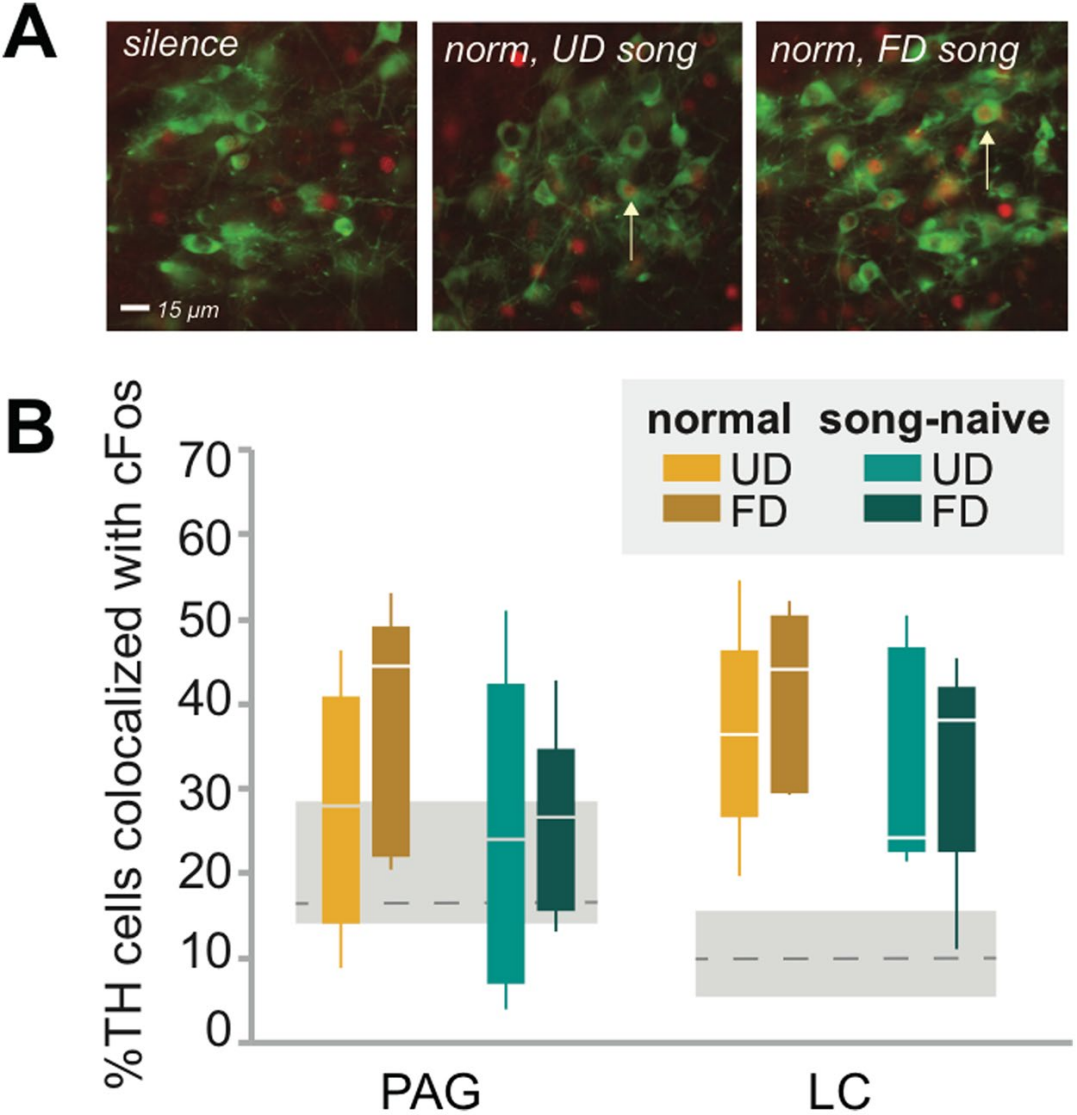
Developmental song exposure and social context do not affect cFOS expression in TH neurons in the LC or PAG. (**A**) Examples of co-localized expression of TH (green) and cFOS (red) in the locus coeruleus (LC) of a normally-reared bird that heard silence (left panel), UD song (middle panel; UD song) and female-directed song (FD; right panel). White arrows indicate examples of co-localized expression. (**B**) Percent of TH neurons that express cFOS in the periaqueductal gray (PAG) and LC. While there was significant song-evoked cFOS expression in TH neurons of the LC (relative to silence; see Results), there were not significant effects of either rearing condition or stimulus context for either the PAG or LC. Box-and-whisker plots are for normally-reared (yellow colors) and song-naïve (green colors) hearing UD (UD; lighter colors) or female-directed (FD; darker colors) songs. Each box spans the interquartile range, horizontal white lines indicate the median and whiskers show the minima and maxima. Levels of cFOS expression in TH neurons for silence controls are plotted as the mean (gray dashed line) +/− standard error (gray boxes). There were no significant effects of rearing condition, stimulus, or an interaction at p < 0.05.

Dopaminergic neurons in the PAG are thought to be involved in arousal^35^, and may also mediate reward responses to opiates^36^. We found that TH neurons of the PAG were not, on average, responsive to song playback relative to silence (p > 0.20; Fig. 2B; Table 1). In addition, neither rearing (p > 0.20), stimulus (p > 0.20) nor the interaction (p > 0.20) contributed significantly to cFos expression in TH-immunoreactive cells. These data highlight the specialized response of TH+ neurons in the caudal VTA relative to other dopamine positive regions of the midbrain.

### Song exposure increases cFos expression in LC regardless of rearing condition or stimulus

Norepinephrine release from the LC has been demonstrated to be significant in modulating attention states and identifying salient stimuli^20,21,37,38^. Similar to previous studies, we found that TH+ neurons in the LC exhibited substantial increases in cFOS expression following song playback (F_(1,25)_ = 31.05, p < 0.0001; Fig. 1C,D, Fig. 2A,B; Table 1^39^). We then we investigated whether rearing (normal versus song-naive), stimulus (FD versus UD song) or their interaction contributed to cFos expression in TH-immunoreactive cells of the LC. While TH neurons of the LC were highly responsive to song playback in general, the expression of cFOS in TH-positive LC neurons was similar between normally-reared and song-naïve females (F_(1,16)_ = 1.52, p = 0.2354), as well as in response to FD and UD song (F_(1,16)_ = 0.28, p = 0.6061). Moreover, there was not a significant interaction between rearing and stimulus (F_(1,16)_ = 0.17, p = 0.6839). These data suggest that although the noradrenergic neurons of the LC are generally responsive to playback of song, cFOS expression in TH neurons of the LC does not appear to be sensitive to rearing condition or to differences between stimuli.

### Developmental auditory experience determines the number of TH-ir neurons in the VTA

**Figure 3:**
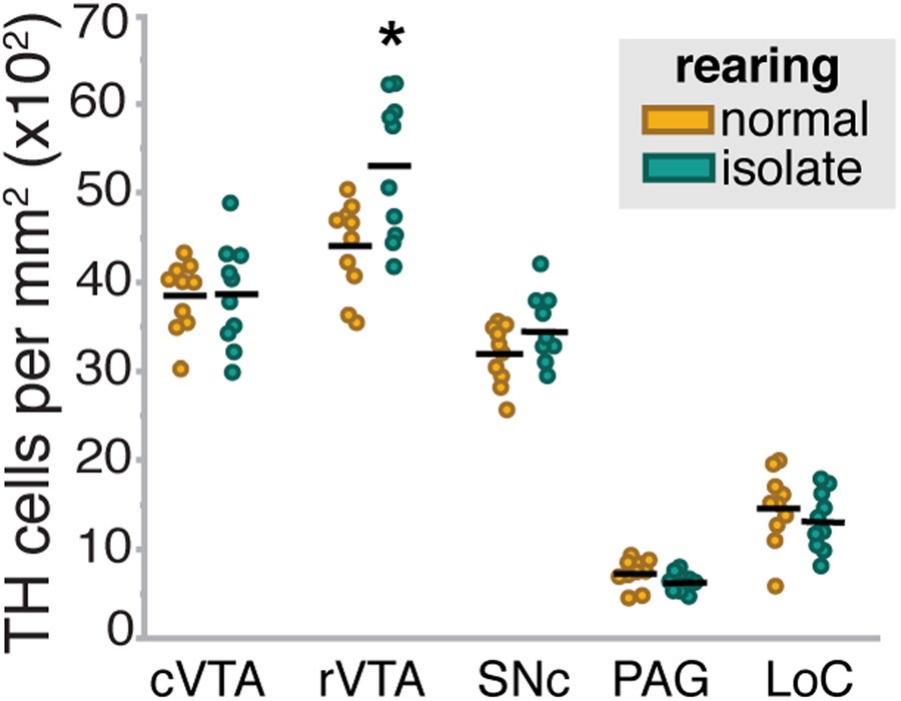
Song-naïve females have a significantly greater density of TH neurons in the rostral VTA. The number of TH-positive neurons per square mm in the caudal VTA (cVTA), rostral VTA (rVTA), substantia nigra pars compacta (SNc), periaqueductal gray (PAG), and locus coeruleus (LC). Points are for normally-reared (yellow) and song-naïve (green) females that heard either female-directed or UD song. While there was not effect of the social context of the stimulus, there was a significant difference between normally-reared and song-naïve females in the rVTA. ^*^Indicates a significant difference at p < 0.05.

We also investigated whether the abundance of catecholamine neurons within specific populations was affected by developmental auditory experience or song playback. In particular, we tested whether the density of neurons expressing TH was differentially affected by stimulus, rearing condition or the interaction within each brain region. In the VTA there were significant effects of region (rostral vs. caudal; F_(2,45.4)_ = 49.9, p < 0.0001), a rearing by region interaction (F_(2,45.4)_ = 8.2, p = 0.0063) and a trend towards an effect of rearing (F_(1,15.9)_ = 4.3, p = 0.0552; Fig. 3; Supplementary Fig. S2). Generally, there were more TH positive neurons in the rostral VTA than the caudal VTA. While the difference in density between the rostral and caudal VTA was apparent in both song-naïve (p < 0.0001) and normally-reared (p = 0.0223) females, the difference was especially pronounced in song-naïve females. In particular, the density of TH neurons in the rostral VTA was significantly higher in song-naïve females compared to normally-reared females (p = 0.0146). The density of TH neurons was not significantly affected, in either region, by stimulus context. Moreover, we did not find significant effects of rearing condition, stimulus or the interaction on TH neuron density in the LC, SNc, or PAG (p > 0.05 for all comparisons).

## Discussion

Acoustic experience during development can shape the tuning and plasticity of auditory systems, and neuromodulators, such as the catecholamines dopamine and norepinephrine, contribute to this sensory plasticity and cortical remodeling in both older and younger individuals^13–15,40^. Less well known, however, is whether developmental auditory experience can have lasting effects on the neuromodulatory populations themselves. Here, we used songbirds to investigate whether sensory experience during development has long-lasting effects on the abundance and sensory responsiveness of catecholamine-synthesizing neurons. We found that in the VTA, but not other catecholamine producing regions of the mid- and hindbrain, developmental exposure to song affected the number of dopamine producing neurons and their modulation by the social context of song in a way that mirrors behavioral preferences for FD courtship song. These data highlight the importance of developmental auditory experience in tuning not only the responses of  auditory cortical regions but also the catecholamine populations that modulate them.

Neurons of the VTA and SNc are a major site of dopamine synthesis critical for motor, sensorimotor, and cognitive behaviors across vertebrates. These neurons broadly respond to sensory stimuli^18,41–45^, and are thought to signal stimulus relevance and incentive value across higher order brain structures^17,18,22^. In female zebra finches, we report greater activation of dopaminergic neurons in the caudal VTA following playback of categorically preferred songs. In particular, we found that cFOS expression in TH neurons of the caudal VTA was higher in response to the preferred, FD song over the less attractive UD song. The specificity of effects in caudal versus rostral VTA is reminiscent of topographical differences in immediate early gene expression in the VTA related to drug sensitivity in rodents^46^ and social modulation of singing-related activity in songbirds^47^. Moreover, these data support growing evidence, in a number of songbird species, that certain songs or song features have significant incentive value to female songbirds^19,48–51^. Interestingly, while a number of studies have implicated dopamine acting in regions like the nucleus accumbens, ventromedial hypothalamus, and medial preoptic area, there has been less evidence of differential responses within the VTA to songs of different incentive value^49,52,53^. Our data, which suggest that dopamine-synthesizing cells of the caudal VTA are sensitive to socially-modulated differences in acoustic features of song, are significant in providing a bridge between VTA signaling and the modulation of preference by dopamine action in sensory, mesolimbic, and hypothalamic regions.

In addition, our data indicate that the incentive value of FD courtship song may be dependent on developmental song exposure. Song-naïve females have been shown to differ in their song preferences relative to normally-reared females^8–10^. Here, we report that whereas normally-reared females exhibit differential expression of cFOS in TH neurons of the caudal VTA in response to FD courtship versus UD song, song-naïve birds show similar, low levels of cFOS expression to both song types. These data parallel behavioral preferences as, unlike normally-reared females, song-naïve females do not show consistent preferences for FD over UD song^9^. Moreover, the lack of differential expression of cFOS in the VTA is reminiscent of a similar effect in the auditory forebrain region NCM where normally-reared but not song-naïve females show greater EGR1 expression in response to FD than UD song. Taken together, these data raise the question of whether deficits in auditory processing contribute to the aberrant behavior and VTA response, or whether deficits in activity in the VTA lead to the aberrant auditory processing. Delineating the independent contributions of neuromodulatory and sensory circuits will be critical for understanding how they each are shaped by developmental auditory experience.

In addition to showing stimulus-dependent changes in activity, midbrain catecholaminergic neurons also show considerable plasticity in the expression of TH. In zebra finches, both males and females show experience-dependent plasticity in TH expression^25,54^. For example, TH expression in the VTA is positively related to the quantity of courtship interactions between co-housed birds and negatively related to the amount of affiliative behavior^52^. We found that rearing condition, but not stimulus, affected the density of TH+ neurons in the rostral VTA. Specifically, song-naïve females had significantly more TH cells/mm^2^ than normally-reared females in the rostral region of the VTA. The dopamine system undergoes substantial growth and pruning over the course of development^55–58^. A growing body of literature points to the role of circuit activity in promoting cell survival^59,60^. It is possible that the lack of song exposure during development in our song-naïve females, a minor but socially significant form of sensory deprivation, could have disrupted normative neuronal pruning or activity-dependent programmed cell death. Alternatively, the greater density of TH neurons in the rostral VTA in our song-naïve females could be due to the stress of single-parent rearing. However, while both developmental maternal separation and chronic stress have been found to affect TH-immunoreactivity in the rat VTA, there is variation in the literature on whether stress increases or decreases midbrain TH expression^61–64^. Future studies manipulating rearing condition and developmental auditory experience will be necessary to tease apart the roles of stress and experience-dependent pruning in the development of the midbrain dopamine system.

Noradrenergic neurons respond to salient signals across vertebrates^37,38,65^, and have been argued to facilitate attentional shifts toward behaviorally relevant information^20,21^. In songbirds, NE can significantly affect auditory processing. Norepinephrine increases the signal to noise of auditory responses in the secondary auditory cortex NCM and NE antagonists as well as neurotoxins that target NE producing neurons minimize the neural and behavioral differences in response to relevant stimuli versus less relevant stimuli, such as conspecific versus heterospecific song^26,27^. Moreover, NE depletion also reduces the number of copulation solicitation displays that female canaries perform in response to sexually-stimulating songs and slows the learning of song discrimination^28,29^. In female zebra finches, song exposure increases IEG expression in TH-cells in the locus coeruleus (LC) relative to silence^39^, an effect which was reproduced in our experiment: all females, both song-naïve and normally-reared, showed increased cFos expression in TH cells of the LC following exposure to song relative to silence. We found no modulation of neural activity or density of TH-ir cells in LC based on whether the song was FD or UD. In addition, the expression of cFOS in TH neurons of the LC was not affected by developmental song exposure. Song-naïve females have been shown to be able to still prefer conspecific song to heterospecific song. Thus, it is intriguing to hypothesize that the ability of the LC to turn attention to specific categories of song, such as conspecific versus heterospecific song, may not require developmental auditory experience. It will be interesting to investigate the degree to which the pattern of LC neuron firing, during song playback *in vivo*, rather than just the sum total of activity changes as measured by cFOS expression, varies for individual conspecific songs.

Vocal communicators are exposed to a wide range of acoustic signals in the environment. The auditory system hierarchically filters acoustic input, preferentially encoding ethologically relevant stimuli and information about their salience, and neuromodulators such as dopamine may contribute to this processing. Female zebra finches are exposed to both courtship and non-courtship songs throughout the lifespan, and exhibit robust preferences for FD songs^9,66^. We found that activity of dopamine neurons in the caudal VTA was sensitive to differences between FD and UD song, but only in females who demonstrate species-typical preferences for FD over UD song. It therefore appears that neural activity during song exposure correlates with behavioral song preference in this area of dopamine synthesis. However, whether the correlations between preference and dopaminergic activity results from dopaminergic inputs to the auditory cortex, or auditory cortical inputs to the VTA is unknown. A growing body of evidence suggests that dopamine release from the VTA might dynamically modulate and reorganize the auditory cortex in favor of certain signals^13,67,68^, and in songbirds TH-ir cells have been found to innervate the auditory forebrain and TH expression is modulated by song playback^24,25,48^. Further investigation of the role of dopamine inputs to the auditory cortex in processing auditory preferences in the zebra finch will be critical for understanding the potential role for catecholamines in the modulation of song preferences.

## Methods

### Animals

All zebra finch females (N = 27, >90 days post-hatch) were kept on a 14:10 light:dark schedule with *ad libitum* access to seed, water, and grit. Lettuce and egg supplements were provided once per week. Bird care and experimental procedures were approved by the McGill University Animal Care Committee and were performed in accordance with the Canadian Council on Animal Care guidelines.

Females were raised in one of two conditions. Normally-reared females were raised in a cage containing both parents and all siblings until 60 days post hatch, then housed in same-sex group cages in a colony that housed both male and female zebra finches. Song-naive females were raised to 60 days with their mothers and sisters in sound-attenuating boxes (TRA Acoustics, Cornwall, Ontario). Fathers were removed from cages five to seven days after hatching, which is early enough to prevent young females from memorizing their song^8^. Male siblings were removed when they were 30–40 days of age. While males this age may produce immature vocalizations (‘babbling’ or ‘subsong’) it is well before they start producing stereotyped song^69^. Around 60 days of age, females were moved to same-sex group cages, which were also contained in sound-attenuated boxes.

### Experimental design

All females were isolated in individual cages in sound-attenuating chambers equipped with a speaker for at least 24 hours before the start of the experiment. On experiment days, females’ lights were turned off beginning at least one hour prior to song playback and remained off until sacrifice to minimize motor activity, including calling, in response to playback and non-specific immediate early gene protein expression. Song-naïve (N = 10) and normally-reared females (N = 10) were exposed to 30 minutes of passive playback of either FD or UD song recorded from one of two males. We used five renditions each of FD or UD song, and played them each 15–18 times in a random sequence at one second intervals at 70 dB. Control females (N = 7) were kept in the dark without playback (‘silence’). Following song playback or silence, females remained in the dark for an additional 45 minutes to allow for protein translation to occur.

### Song stimuli

Stimuli were FD and UD songs from two different males that were unfamiliar and unrelated to the experimental females. Songs were recorded as previously described^9,66^. Briefly, males were housed individually in a cage inside a soundbox equipped with a microphone and video camera. Vocalizations were recorded using custom written sound-activated recording software (44.1 kHz). To collect FD song, we introduced a cage containing a female into the soundbox next to the male’s cage and monitored the male’s behavior on a video monitor. During courtship song performance, males orient toward the female, fluff the body feathers while flattening feathers on top of the head, hop, dance, and beak wipe^66,70–72^. Only song renditions where males performed at least two of the above courtship components were considered to be FD songs. Males performed one to two bouts of song for each female presentation. After removing the female, we waited up to 10 minutes before reintroducing the female in order to collect interleaved bouts of UD song. We also recorded an additional one hour of UD song before the first and after the last female presentation on each recording day. Males were recorded in the morning and were often recorded over multiple days in order to ensure a sufficient number of FD song bouts to use for stimuli.

We selected five renditions of FD and UD song to be used as stimuli. We selected song renditions that represented the mean and range of variation in song duration for a particular male. In particular, we chose songs that exemplified the mean, minimum, and maximum number of introductory notes and motifs. Songs were verified to be free of noise artifacts, including female calls, then bandpass filtered, normalized by the maximum amplitude and saved as a wav file (44.1 kHz).

### Immunocytochemistry

Females were acutely anesthetized through isoflurane inhalation and transcardially perfused with 25 ml of saline, containing 12 mg of heparin, followed by 150 ml of 4% paraformaldehyde. Once brains were removed from the skull, they were stored for 4–12 hours in 4% paraformaldehyde and then transferred to 30% sucrose for cryoprotection at 4 C. Brains were cut in 40 μm sagittal sections and kept in 0.025 M phosphate-buffered saline containing sodium azide before starting immunocytochemical procedures.

We performed double-label immunocytochemistry, which allowed us to target catecholamine-synthesizing cells, and to quantify neuronal activity using immediate early gene expression induction. We performed immunocytochemistry in batches (n = 5 batches) in which we processed every third section from a bird from each experimental group: (1) song-naïve birds that heard FD song; (2) song-naïve birds that heard UD song; (3) normally-reared birds that heard FD song; and (4) normally-reared birds that heard UD song. Females included in each batch all heard the songs of the same male. We also included silence control birds in all immunocytochemical batches to assess whether catecholamine-producing regions were song-responsive.

All immunocytochemical procedures were performed as previously described^9,23,47,73^. Briefly, brain sections underwent 3 × 10-min rinses in 0.025 M PBS followed by a 1-h incubation in 5% donkey serum and 0.3% Triton X-100. Sections were then incubated for 48-h at 4 °C in primary antibody: rabbit anti-cFOS (1:1000 dilution; Cat# SC-253; Santa Cruz Biotechnology, Santa Cruz, CA, USA) and sheep anti-tyrosine hydroxylase (TH; n = 6 batches; 1:1000 dilution; Cat# NB300-110, Novus Biologicals, Littleton, CO, USA). Sections were then washed (3 × 10-min) and incubated for 2-h at room temperature in donkey anti-rabbit secondary antibody conjugated to Alexa Fluor 594 (for cFOS; 5 μl/ml; Life Sciences, Burlington, ON, Canada) and donkey donkey anti-sheep secondary antibody conjugated Alexa Fluor 488 (for TH; 3 μl/ml; Life Sciences). Following another wash (3 × 10-min), sections were mounted and cover-slipped (ProLong Gold Antifade Reagent, Life Sciences) on chromium-aluminium subbed slides.

Four regions of interest were imaged: VTA, PAG, SNc, and LC. Regions were identified using TH expression based on previous descriptions in the literature^47,74^. We imaged the PAG, a tight cluster of parvocellular TH-positive neurons near the caudal edge of the midbrain, on sections medial to or including the VTA. In our tissue samples (composed of every third sagittal section spanning both hemispheres) PAG was clearly visible in 2.9 ± 0.2 sections/bird (mean ± SEM, range 1–5 sections/bird). We imaged medial sections of VTA where it clearly bordered the rostral edge of the midbrain. We used these conservative boundaries to avoid any ambiguity between the lateral VTA and the medial neurons of SNc. Within sections where the VTA bordered the rostral edge of the midbrain, we divided the VTA into rostral and caudal portions based on previous work which has described two discrete neuronal populations with different functionalities [40, 62]. We imaged the rostral VTA, which contains large densely-packed cell bodies, at the rostral edge of the midbrain in 3.6 ± 0.2 sections/bird (range: 2–5). The caudal VTA, which consists of smaller, more sparse neurons than the rostral population, was imaged where the caudal tip extends away from the center of VTA (3 ± 0.2 sections/bird, range 1–5; Fig. 1B). Moving laterally, the rostral population of TH-positive cells in the VTA disappear and the caudal population becomes more sparse, or, in some instances, disappear completely before returning as a dense population of neurons with larger cell bodies (SNc). The SNc extends quite far laterally, and we imaged TH neurons where they were large and densely packed throughout the wide extent of the nucleus (9.4 ± 0.4 sections/bird; range 5–13) Caudal to both the VTA and/or the SNc, we imaged LC, the densely packed parvocellular cluster of TH-neurons arranged in a circular fashion, in 3.1 ± 0.2 sections/bird (range 1–5).

For all regions, compound images overlaying TH-immunoreactive cells bodies and cFos-immunoreactive nuclei were taken for both hemispheres of each region (rostral VTA, caudal VTA, PAG, LC, and SNc) with the 20x objective of a Zeiss Axio Imager upright microscope and an AxioCam MRm Zeiss camera (Carl Zeiss, Germany). Exposure settings were standardized within batches by obtaining the average optimal exposure settings of tissue across each bird of a batch. Using Zen microscopy software (Carl Zeiss, Germany), TH-labelled neurons and cFos-labelled nuclei were summed for each region separately by channel. Where TH-labelled neurons and cFos-labelled nuclei co-occurred, colocalization was verified in dual-channel view. The total number of TH-labelled neurons and cFos-labelled nuclei were summed for each region, and the sum was divided by the area of the counting window in order to present them as a density (the number of neurons/mm^2^). We also summed the number of TH-neurons colocalized with cFos-nuclei and the percent of TH-cells colocalized with cFos for each region. Images were collected and counts were performed by an individual blind to bird ID and stimulus condition.

In order to confirm colocalization of cFos within TH neurons, a subset of sections in the caudal VTA were re-imaged using a Zeiss LSM 710 confocal microscope with a multiline argon laser (458; 488; 514 nm, 25 mW) and a Helium/Neon laser (594 nM, 1.2 mW) on a Zeiss Axio Observer.Z1 fully motorized inverted microscope. Image stacks were collected through the z-axis at 2 micron steps, resulting in 7 to 10 images/stack. In these images, the total number of cFos-labelled nuclei and TH-labelled neurons were summed separately per channel using maximum intensity projections generated in ImageJ [76]. Where cFos and TH were co-labelled, colocalization of the cFos-positive nucleus within the TH-cell body was verified in individual z-slices. Images were collected and counts were performed by an individual blind to bird ID and stimulus condition

### Analysis

We first tested whether catecholamine-synthesizing cells within each of the five regions respond to song exposure. For this, we computed the average percent of TH neurons expressing cFOS for each region for each bird. For each region, we compared the mean percent of TH neurons expressing cFOS for all females exposed to song versus all silence control females using sound condition (silence or song) as an independent variable, and batch and bird ID nested within batch as random variables.

To analyze the effects of rearing and stimulus context on cFos expression in TH-positive cells across regions and birds, we computed the mean density of TH neurons and the mean percent of TH neurons colocalized with cFos for each hemisphere in each region imaged (rostral VTA, caudal VTA, SNc, PAG, LC) for each bird.

For the VTA, which forms a complex of TH-positive neurons in the mesencephalon, we used a full-factorial statistical model with song stimulus (FD vs. UD), rearing (song-naïve vs. normal), region (rostral VTA vs. caudal VTA), hemisphere (left vs. right) and all possible interactions as independent variables. We included batch and bird ID nested in batch as random variables and separately analyzed the mean percent of TH cells co-localized with cFos and the mean density of TH-positive neurons as dependent variables.

We also analyzed the density of TH-positive neurons and the expression of cFos in TH cells the LC in the hindbrain and the SNc and PAG in the midbrain. Within each region, we applied a full factorial statistical model with song stimulus (FD vs. UD), rearing (song-naïve vs. normal), hemisphere (left vs. right) and all possible interactions as independent variables. Batch and bird ID nested in batch were included as random variables. We separately analyzed the mean percent TH cells co-localized with cFos and the mean density of TH-positive neurons (mean number of cell bodies/mm^2^) in each region as dependent variables.

We compared the percent colocalizations of cFos and TH-neurons obtained for the caudal VTA using both confocal stacked image and fluorescent single image microscopy methods. We applied a paired t-test to the mean percent of colocalized neurons for the confocal stacked image and the single image. We also tested a model with rearing and stimulus as independent variables, the percent of colocalization in the confocal stacked images as a dependent variable, and batch and batch nesting into female ID as random variables.

Statistical analyses were completed using JMP Statistical Processing Software (SAS, Cary, NC, USA) or custom-written Matlab code (Mathworks, Natick, MA).

For all statistical models we used a restricted maximum likelihood approach with unbounded variance components. We used least-squared Tukey’s HSD, within the mixed model, for all post-hoc tests and set α < 0.05 for all tests unless otherwise noted.

The datasets generated and analyzed during the current study are available on request.

## Electronic supplementary material

Supplementary Figures
