# Supplementary material for: Developmental auditory exposure shapes responses of catecholaminergic neurons to socially-modulated song
Source: Sci Rep. 2018 Aug 6;8:11717. doi: 10.1038/s41598-018-30039-y (PMC6079043; doi:10.1038/s41598-018-30039-y)
Supplement: Supplementary file 1 — Supplementary Figures [file 41598_2018_30039_MOESM1_ESM.pdf]

Developmental auditory exposure shapes responses of catecholaminergic neurons to socially-modulated song

Helena J. Barr[1,2] and Sarah C. Woolley[1,2,3]

[1] Integrated Program in Neuroscience, McGill University, Montreal, QC, Canada

[2] Center for Research on Brain, Language, and Music, McGill University, Montreal QC, Canada

[3] Department of Biology, McGill University, Montreal, QC, Canada

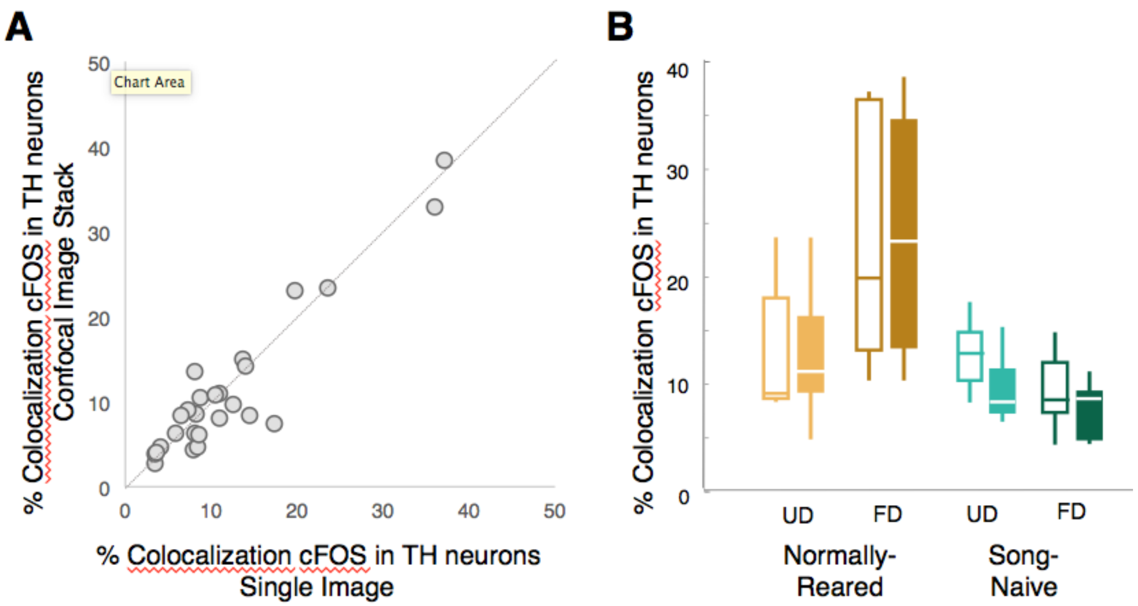

Supplementary Figure S1 *Correspondence between imaging methods for measurements of colocalization*

A) The percent of TH neurons expressing cFOS as measured on a single image compared to the percent measured on a confocal image stack. There is a high degree of correspondence in the percent of colocalized neurons between the two methods (points are on or near the line of unity). B) The overall effect of greater colocalization of TH and cFOS in the caudal VTA of normally-reared birds that heard FD song compared to normally-reared birds that heard UD song or song-naïve birds is apparent using both the single image method (open bars) and confocal image stack (filled bars). Box-and-whisker plots are for normally-reared (yellow colors) and song-naïve (green colors) hearing UD (UD; lighter colors) or female-directed (FD; darker colors) songs. Each box spans the interquartile range, horizontal lines indicate the median and whiskers show the minima and maxima. Within each imaging method, the percent of TH neurons expressing cFOS in normally-reared females that heard FD song is significantly greater ($p<0.05$) than in normally-reared females that heard UD song or in either playback condition of song-naïve females

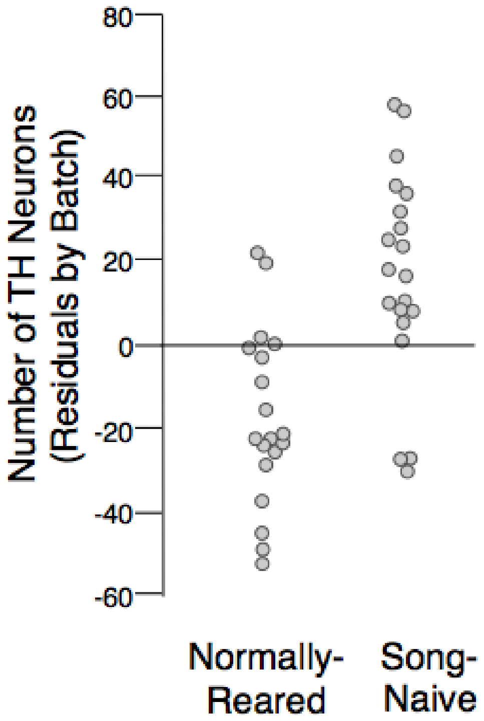

Supplementary Figure S2 *Rearing condition significantly affects TH neuron density in the rostral VTA*
Differences between the batches had the potential to obscure group differences in plots of the number of TH neurons. To better depict differences between rearing conditions, we calculated the residuals controlling for batch [Number of TH cells – mean (Number of TH cells per batch)] and have plotted them here. Points are the residuals for the average number of TH neurons in each hemisphere, for each bird. These data highlight the significantly lower density of TH neurons in normally-reared females than in song-naïve females.
